# Plasma Extracellular Vesicle Characteristics Correlate with Tumor Differentiation and Predict Overall Survival in Patients with Pancreatic Ductal Adenocarcinoma Undergoing Surgery with Curative Intent

**DOI:** 10.3390/jpm11020077

**Published:** 2021-01-28

**Authors:** David Badovinac, Katja Goričar, Hana Zavrtanik, Miha Petrič, Teja Lavrin, Nina Mavec, Vita Dolžan, Aleš Tomažič, Metka Lenassi

**Affiliations:** 1 Department of Abdominal Surgery, University Medical Centre Ljubljana, Zaloška 7, 1000 Ljubljana, Slovenia; david.badovinac@kclj.si (D.B.); hana.zavrtanik@kclj.si (H.Z.); miha.petric@kclj.si (M.P.); 2 Institute of Biochemistry and Molecular Genetics, Faculty of Medicine, University of Ljubljana, Vrazov trg 2, 1000 Ljubljana, Slovenia; katja.goricar@mf.uni-lj.si (K.G.); teja.lavrin@mf.uni-lj.si (T.L.); nina.mavec@yahoo.com (N.M.); vita.dolzan@mf.uni-lj.si (V.D.); 3 Department of Surgery, Faculty of Medicine, University of Ljubljana, Zaloška 7, 1000 Ljubljana, Slovenia

**Keywords:** pancreatic cancer, extracellular vesicles, nanoparticle-tracking analysis, tumor differentiation, overall survival, plasma biomarkers, liquid biopsy

## Abstract

Better preoperative characterization of patients with pancreatic ductal adenocarcinoma (PDAC) would aid in treatment optimization. Extracellular vesicles (EV) are promising, largely unexplored biomarkers in PDAC. This study aimed to evaluate if plasma EV characteristics are associated with PDAC clinical characteristics and overall survival (OS). The prospective cohort included 34 PDAC patients undergoing surgery with curative intent. Patient data and plasma samples were collected preoperatively, intraoperatively and one month postoperatively. Small plasma EV (sEV) concentration and size were determined by nanoparticle-tracking analysis. A Mann–Whitney test, Spearman’s rho and Cox regression were used in statistical analysis. Preoperatively, patients with poorly differentiated tumors had significantly larger plasma sEVs when compared to patients with well/moderately differentiated tumors (mean diameter 176.9 vs. 149.2 nm, *p* = 0.021), the sEV size even enabling discrimination of the two groups (AUC = 0.742, 95% CI = 0.560–0.923). Plasma sEV characteristics were also a predictor of OS in multivariable analysis. Patients with a more than 33.8% increase in sEV concentration after one month had 7.2 months shorter median OS (*p* = 0.002), while patients with a more than 28.0% decrease in sEV size had 9.2 months shorter median OS (*p* = 0.045). Plasma sEV concentration and size correlate with tumor differentiation and may predict OS in PDAC patients. In the future, plasma sEV characteristics could contribute to improved patient stratification for optimized treatment.

## 1. Introduction

The overall 5 year survival of less than 10% makes the pancreatic ductal adenocarcinoma (PDAC) one of the deadliest cancers known, with the incidence rising in the developed world [1,2,3]. Due to late onset of symptoms, 80% of patients are diagnosed with advanced, unresectable stage of disease [1,3,4]. Current management strategies for resectable disease employ upfront radical surgery followed by adjuvant treatment, while for borderline resectable PDAC, neoadjuvant therapy followed by resection is proposed [4,5,6]. To improve survival, important advances in neoadjuvant and adjuvant regimens were achieved recently [4,5,7], with huge efforts dedicated to identifying specific biomarkers that would enable earlier diagnosis and more optimal treatment of PDAC [8]. While biomarkers for early detection are still lacking [9], preoperative identification of patients with advanced disease or poor prognosis, despite tumor resectability, could aid in treatment optimization. Surgery could be avoided or postponed in these patients and systemic treatment immediately applied, possibly resulting in improved survival or at least quality of life [10,11]. Among preoperatively obtainable characteristics, tumor differentiation [12,13] and serum carbohydrate antigen 19-9 (CA 19-9) levels are associated with survival and tumor resectability [10,14]. As tissue histological grading from preoperative fine needle aspiration/biopsy is invasive and unreliable and serum CA 19-9 has its own limitations [8,15], non-invasive liquid biopsy reflecting tumor heterogeneity could importantly contribute to improved patient stratification for optimized therapy [16].

Liquid biopsy is a test performed on biofluid samples, most commonly blood, in order to diagnose and monitor various diseases, among which several cancers have been in the spotlight. Only 1% of the literature on liquid biopsy in cancer focuses on PDAC [17]; still, meta-analysis of this supports the use of liquid biopsy as surrogate for tissue biopsy [18]. By obtaining tumor-derived material from peripheral blood of PDAC patients, genetic alterations (e.g., *KRAS*) reflecting tumor heterogeneity were identified in circulating tumor DNA (ctDNA), while analysis of circulating tumor cells (CTCs) and ctDNA showed potential for monitoring treatment outcome and disease progression (reviewed in [16,18,19]). Still, studies on the use of CTCs and ctDNA as PDAC biomarkers are not conclusive; therefore, novel approaches based on ctDNA methylation profiling or fragmentation patterns were proposed [20,21]. DNA methylation can help determine the tissue origin of ctDNA, as it is highly tissue specific but consistent among different individuals and cancer patients [20,21]. Another promising and still largely unexplored liquid biopsy biomarkers in PDAC are extracellular vesicles (EVs).

EVs are a heterogeneous population of membrane bound particles, which are shed from all cell types and accumulate in all body fluids, including blood and pancreatic juice [22,23,24]. According to their size and site of formation, EVs are subdivided into exosomes, microvesicles and apoptotic bodies. In PDAC, EVs are implicated in the pathogenesis, local progression, metastasis, immune evasion and intercellular communication [24]. EVs molecular composition and biophysical properties mirror the (patho)physiological state of the cell of origin and thus they have great potential for human diagnostics and therapeutic applications [22,23]. Importantly, the multiple distinct biological materials contained within the EV can enable improved sensitivity and specificity of combined EV biomarkers [25,26]. EV DNA [27], miRNA [28] and protein [29,30] cargo were shown to correlate with disease stage and survival in PDAC patients, and EVs were also studied as therapeutic targets or agents [24]. EV concentration itself could also be used as a biomarker for PDAC, since several cancers are characterized by a remarkable increase in total plasma levels of EVs [22], but this has not been specifically studied to date.

Our study aimed to evaluate if small plasma EV (sEV) concentration and size are associated with PDAC clinical characteristics and patients’ overall survival (OS) in a prospective cohort of PDAC patients undergoing surgery with curative intent. We showed that patients who underwent tumor resection did not differ significantly from patients with solely surgical exploration in studied clinical and EV characteristics. Importantly, however, patients with poorly differentiated tumors had significantly larger plasma sEVs when compared to patients with well/moderately differentiated tumors. Furthermore, plasma sEV concentration and size were significant predictors of OS after adjustment for clinical variables.

## 2. Materials and Methods

### 2.1. Study Design and Data Collection

Patients with definite or suspected diagnosis of PDAC were eligible for inclusion in this prospective cohort study, and they all underwent surgery with curative intent from 1 January to 30 September 2018, at the Department of Abdominal Surgery, University Medical Centre Ljubljana, Ljubljana, Slovenia. Depending on the intraoperative assessment of the extent of the disease, patients underwent either surgical resection or exploration without resection. If diagnosis of PDAC was not confirmed by histopathological examination of the resected tissue or intraoperative biopsy obtained at exploration, patients were excluded from the study. Patients who received neoadjuvant treatment were not eligible for study enrollment. The study was conducted in accordance with the Declaration of Helsinki and approved by the Republic of Slovenia National Medical Ethics Committee (Study No. 0120-155/2016-2, KME 106/03/16). Written informed consent was obtained from all subjects prior to their enrollment. 

Patient data were collected before, during and one month after surgery. Patients’ vital status was determined on 24 May 2019. Data included patient demographics, American Society of Anesthesiologists (ASA) score, smoking status, alcohol consumption, body mass index (BMI), tumor size on preoperative computed tomography scan and adjuvant chemotherapy if applicable. Laboratory report included white blood cell (WBC) count, C-reactive protein (CRP), CA 19-9 and carcinoembryonic antigen (CEA). Pathology report included surgical resection status (R0, R1 and R2), tumor differentiation (well, moderate or poor) and tumor TNM classification. Any missing patient data due to follow-up non-attendance (poor health, disease progression and death) are clearly indicated.

Blood samples for EV isolation were collected immediately before surgery and again one month after surgery in K2-EDTA collection tubes (6 mL). Samples were processed within 4 h by centrifugation at 2500× *g* for 10 min at 4 °C and plasma aliquots stored at –80 °C. Any sample exclusion due to visually positive hemolysis is clearly indicated. 

### 2.2. Small EV Isolation from Blood Plasma

One milliliter of plasma was thawed on ice and centrifuged at 10,000× *g*, for 20 min at 4 °C. Next, supernatant was diluted to 9 mL with phosphate-buffered saline (PBS) and pipetted over 2 mL of 20% sucrose in 13 mL tubes. After centrifugation at 100,000× *g*, for 2 h 15 min at 4 °C (MLA-55 in Optima MAX-XP, Beckman Coulter), supernatant was aspirated, the pellet suspended in 60 μL of PBS and aliquots stored at −20 °C until analysis. The described procedure enables isolation of sEVs and exclusion of most lipoproteins from plasma, as determined for 10 healthy volunteers by electron microscopy, nanoparticle-tracking analysis, asymmetrical flow field-flow fractionation connected to detectors and miRNA expression analysis [31,32]. 

### 2.3. Quantification of sEV Concentration and Size

Small EV concentration and size were determined by nanoparticle-tracking (NTA) analysis using the NanoSight NS300 instrument (488 nm laser) connected to an automated sample assistant (both Malvern Panalytical). Samples were diluted 200 and 400 times in PBS and recorded five times at camera level 14. Raw data were analyzed by the NanoSight NTA 3.3 program at the following settings: detection threshold 5, water viscosity, temperature 25 °C, automatic settings for minimum expected particle size and blur, and minimum track length 10. Output data were expressed as sEV concentration, that is, the number of particles per 1 mL plasma, and sEV size, that is, the mean, modal and median hydrodynamic diameter in nm. Coefficients of variation for sEV concentration and size measurements were 5% and 2–6%, respectively. Relative change was defined as the difference of sEV concentration or size values one month after and before surgery, divided by its value before surgery.

### 2.4. Statistical Analysis

All analyses were performed using IBM SPSS Statistics, version 21.0 (IBM Corporation, Armonk, NY, USA). Continuous and categorical variables were described using the median with interquartile (25–75%) range and frequencies, respectively. A nonparametric Mann–Whitney test and Fisher’s exact test were used to compare the distribution of continuous variables and categorical variables among different patient groups, respectively. Spearman’s rho correlation coefficient (ρ) was used to assess correlations between continuous variables. In survival analysis, Cox regression was used to calculate hazard ratios (HRs) and the corresponding 95% confidence intervals (CIs). Clinical variables used for adjustment in multivariable survival analysis were selected among all reported clinical variables using stepwise forward conditional selection. Kaplan–Meier analysis was used to calculate median survival and follow-up times. OS was defined as the time from surgery to death from any cause. A receiver operating characteristic (ROC) curve was used to determine the area under the curve (AUC) and cutoff with the highest sum of specificity and sensitivity. All statistical tests were two sided with the level of significance set to 0.05.

## 3. Results

### 3.1. Patient Characteristics

Characteristics of 34 included patients are presented in Table 1. Curative resection was achieved in 11 patients (four R0 ≤ 1 mm, 22.2%; seven R0 > 1 mm, 38.9%), resection margins were microscopically positive in four patients (R1; 22.2%) and two had macroscopic residual tumor (R2; 11.1%). For one patient, resection margins were not described. Seven patients without resection had stage III and nine stage IV disease; among patients who underwent resection, two had stage IIA, 13 stage IIB, one stage III and two stage IV disease. No significant difference was observed in the clinical characteristics between patients with or without tumor resection, with the exception of distant metastases, which were less likely to be present in patients with tumor resection (*p* = 0.009).

### 3.2. Patients’ Plasma sEV Characteristics

Plasma sEV concentration and size were determined immediately before (*n* = 34) and one month after surgery (*n* = 27, 79.4%) (Table 2). No statistically significant difference in sEV concentration was found between patients with and without resection. On the other hand, larger sEVs were detected before surgery in patients undergoing resection compared to patients without resection (modal diameter 144.0 vs. 132.2 nm, *p* = 0.039). One month after surgery, sEVs were still larger in patients undergoing resection, but the difference was no longer statistically significant (modal diameter 136.5 vs. 124.8 nm, *p* = 0.286).

Since mostly no statistically significant differences in patients’ clinical and sEV characteristics with regard to tumor resection were found, all further analyses were performed on the entire study cohort. Higher sEV concentration correlated with smaller sEVs (ρ = −0.363, *p* = 0.035; ρ = −0.387, *p* = 0.024; ρ = −0.366, *p* = 0.034 for mean, modal and median diameter, respectively). Additionally, relative increase in sEV concentration at one month after surgery was associated with a relative decrease in sEV size (ρ = −0.570, *p* = 0.002; ρ = −0.573, *p* = 0.002; ρ = −0.568, *p* = 0.002 for mean, modal and median diameter, respectively). Relative change in EV characteristics was defined as the difference of sEV concentration or size values one month after and before surgery, divided by its value before surgery. 

### 3.3. Association between Patients’ Clinical and Plasma sEV Characteristics 

Association between patients’ clinical and plasma sEV characteristics are presented in Table S1. Increased inflammatory parameters, such as CRP and WBC count, tended to be associated with smaller sEVs (modal diameter), but the association did not reach statistical significance for WBC count (ρ = −0.376, *p* = 0.031 for CRP levels; ρ = −0.342, *p* = 0.051 for WBC count). Patients with higher ASA score had larger sEVs (mean diameter, *p* = 0.038), while other clinical characteristics were not significantly associated with sEV characteristics. Preoperatively evaluated tumor size or presence of distant metastases were thus not associated with sEV concentration or size (see Table S1).

**Figure 1:**
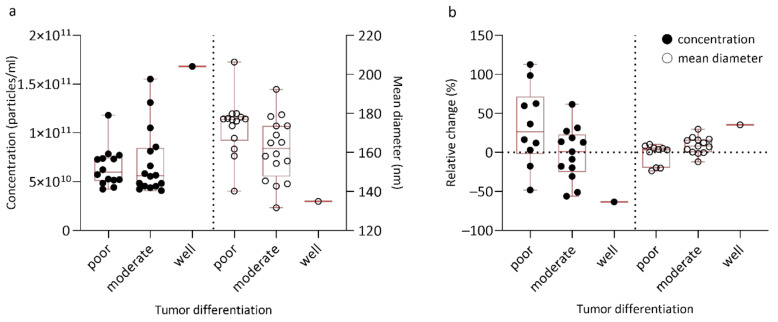
Box plot representing small EV concentration and size in regard to tumor differentiation at different timepoints: (**a**) before surgery; (**b**) one month after surgery expressed as relative change. Relative change in EV characteristics was defined as the difference of small plasma EV (sEV) concentration or size values one month after and before surgery, divided by its value before surgery.

Small EV concentration and size in regard to tumor differentiation are presented in Table 3 and Figure 1a. Importantly, before surgery, sEVs were significantly larger in poorly differentiated tumors when compared to well/moderately differentiated tumors (mean diameter 176.9 vs. 149.2 nm, *p* = 0.021 and median diameter 159.9 vs. 149.2 nm, *p* = 0.023). Lower sEV concentration tended to be associated with decreasing tumor differentiation (*p* = 0.984) (Figure 1a), the only patient with well differentiated tumor having the highest sEV concentration. At one month after surgery, a trend towards a higher (more positive) relative change in sEV concentration and lower (more negative) relative change in sEV size was observed for decreasing tumor differentiation (Figure 1b).

Using ROC curve analysis, we determined cutoff values for sEV characteristics to discriminate between poorly and well/moderately differentiated tumors (see Table S2). At the cutoff value of 173.55 nm for mean diameter before surgery, sensitivity for predicting poor differentiation was 0.765 and specificity 0.714, with an AUC of 0.742 (95% CI = 0.560–0.923, *p* = 0.022). Similarly, at the cutoff value of 158.85 nm for the median diameter before surgery, sensitivity for predicting poor differentiation was 0.824 and specificity 0.643, with an AUC of 0.736 (95% CI = 0.534–0.917, *p* = 0.025).

### 3.4. Patients’ Clinical and Plasma sEV Characteristics and Overall Survival

The median OS of study patients was 9.6 (5.2–15.9) months, with a follow-up time of 12.5 (11.3–14.3) months. At the time of vital status data collection, 14 (41.2%) patients were still alive. In univariable analysis, higher age and CA 19-9 before surgery were associated with shorter OS (HR = 1.08, 95% CI = 1.03–1.14, *p* = 0.004 and HR = 1.00, 95% CI = 1.00–1.00, *p* = 0.007, respectively), while adjuvant chemotherapy improved OS (HR = 0.20, 95% CI = 0.08–0.54, *p* = 0.001). If tumor resection was performed, patients had slightly longer OS compared to patients with exploration only, but the difference did not reach statistical significance (HR = 0.45, 95% CI = 0.18–1.11, *p* = 0.082). In a multivariable regression model, adjuvant chemotherapy and CRP before surgery were the only significant predictors of OS (HR = 0.11, 95% CI = 0.04–0.37, *p* < 0.001 and HR = 1.04, 95% CI = 1.01–1.06, *p* = 0.002, respectively).

**Figure 2:**
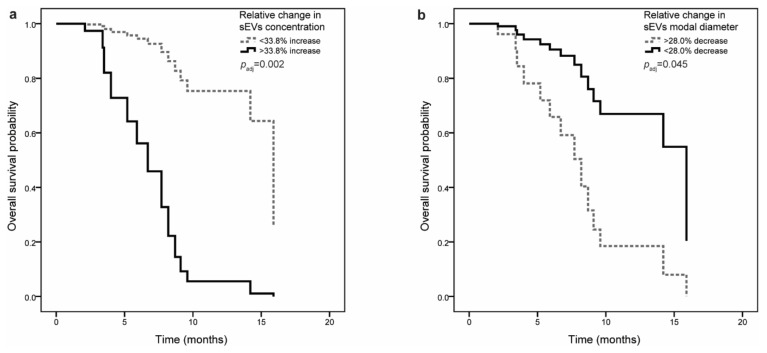
Multivariable Cox regression analysis of overall survival in pancreatic ductal adenocarcinoma (PDAC) patients (*n* = 34): (**a**) Association of relative change in small EV concentration with overall survival. If sEV concentration increased by more than 33.8%, patients had shorter overall survival. (**b**) Association of relative change in small EV in modal diameter with overall survival. If sEV modal diameter decreased by more than 28.0%, patients had shorter overall survival.

Small EV concentration or size before surgery were not associated with OS. However, when adjusted for CRP levels before surgery and for adjuvant chemotherapy, shorter OS was observed in patients with higher (more positive) relative change in sEV concentration (HR = 1.25, 95% CI = 1.05–1.50, *p* = 0.015) and lower (more negative) relative change in sEV size (modal diameter; HR = 0.74, 95% CI = 0.57–0.95, *p* = 0.019) (Table 4). Patients were next stratified according to the cutoff values for sEV characteristics (see Table S2), and the association with OS was evaluated. If sEV concentration increased by more than 33.8%, patients had shorter OS (8.7 (3.4–8.7) months compared to 15.9 (7.7–15.9) months). Even though the association with OS was not significant in univariable analysis (HR = 2.67, 95% CI = 0.84–8.45, *p* = 0.095), relative change in sEV concentration was a significant predictor of OS after adjustment for clinical variables (HR = 10.21, 95% CI = 2.33–44.67, *p* = 0.002) (Figure 2a). If sEV size (modal diameter) decreased by more than 28.0%, patients had shorter OS (6.7 (2.1–7.7) months compared to 15.9 (8.2–15.9) months) both in univariable and multivariable analysis (HR = 0.18, 95% CI = 0.05–0.67, *p* = 0.010 and HR = 0.24, 95% CI = 0.06–0.97, *p* = 0.045, respectively) (Figure 2b).

## 4. Discussion

This study is to our knowledge the first to correlate sEV concentration and size to tumor differentiation and OS in PDAC patients undergoing surgery, and only a few similar studies can be found in other cancers [33,34,35,36]. Patients who underwent tumor resection did not differ significantly from patients with solely surgical exploration in studied clinical characteristics, sEV characteristics and OS. Importantly, however, patients with poorly differentiated tumors had significantly larger plasma sEVs before operation when compared to patients with well/moderately differentiated tumors, the sEV size even enabling discrimination of the two groups. Furthermore, plasma sEV characteristics were a significant predictor of OS after adjustment for clinical variables, with shorter OS observed in patients with higher relative change in sEV concentration and lower relative change in sEV size in one month after surgery.

As shown here and by others, certain PDAC patients undergoing resection have OS similar to those with advanced disease [15], with tumor differentiation being an important prognostic factor of resectability and OS [11,12,15]. Poorly differentiated tumors are associated with worse outcome, but the parameter is routinely obtained intraoperatively by tumor biopsy or resection, while the preoperative endoscopic ultrasound-guided fine-needle biopsy lacks accuracy [15]. Consequently, patients can be exposed to surgical overtreatment with associated complications and suboptimal PDAC management, as those with poorly differentiated PDAC are most likely to benefit from neoadjuvant therapy or immediate systemic treatment initiation [7]. Serum CA 19-9, an alternative preoperative parameter correlating with resectability and OS [10,14], similarly lacks in specificity (elevated in various cancers and benign diseases) and sensitivity (Lewis antigen-negative individuals) [8].

Importantly, our study demonstrated that preoperative plasma sEV size is associated with tumor differentiation. Previously, larger sEVs were associated with metastatic compared to non-metastatic PDAC [27]. We additionally showed that sEVs with a mean diameter >173.55 nm or a median diameter >158.85 nm could discriminate patients with poorly differentiated tumors from those with well/moderately differentiated tumors, yet with modest AUC and limited sensitivity and specificity (0.765 and 0.714, 0.824 and 0.643, respectively). To improve the clinical utility of sEV size, further larger studies combining EV characteristics and other molecular biomarkers, such as EV cargo or CA 19-9, are needed, as composite biomarkers are more likely to have a better predictive ability [25,26]. Alternatively, classification based on sEV size could be used complementary to endoscopic ultrasound-guided fine-needle biopsy findings to improve preoperative assessment of tumor histological grade and thus aid in the personalized treatment of PDAC. Plasma EVs better represent tumor heterogeneity and real-time state of the disease [16,25]. EV concentration and protein levels were similarly associated with tumor differentiation in colorectal and glioma cancer, respectively [33,34,35].

In our study cohort, previously recognized parameters associated with OS in PDAC were identified, such as age, CA 19-9, CRP and adjuvant chemotherapy [11,13,14], but we additionally showed that changes in sEV concentration and size are significant predictors of OS after adjustment for clinical variables. Higher relative change in sEV concentration and lower relative change in sEV size in one month after surgery were associated with shorter OS. Patients with a more than 33.8% increase in sEV concentration had 7.2 months shorter median OS than patients below this cutoff value, while patients with a more than 28.0% decrease in sEV size had 9.2 months shorter median OS than patients above this cutoff value. Similarly, higher plasma sEV [27] or serum glypican-1-positive exosome [30] levels predicted worse OS in localized and metastatic PDAC, while a greater decrease in serum glypican-1-enriched exosomes was proposed to improve OS in all stages of PDAC [29]. Supporting the relevance of high EV levels in predicting OS in PDAC, plasma EVs with >5% exosome *KRAS* mutant allele fraction, high miR-4525, miR-451a, miR-222, miR-21 or circ-PDE8A expression were all associated with worse OS in previous studies [37,38,39,40].

EVs have been shown to be an important prognostic factor in various cancers [23,41], with high EV concentration or small EV size shown to be predictive of less time to relapse and/or worse OS in colorectal, prostate, esophageal and lung cancers [34,36,42,43,44]. High plasma EV concentration in cancer can to some extent be associated with tumor burden [28,45,46]; however, inflammation and response to systemic treatment could also contribute [47,48]. The observed increase in plasma EVs might be connected to physiological factors, such as hypoxia, autophagy or stress, which are often altered in tumors [49]. In our study, there was no significant impact of tumor size or presence of metastases on EV characteristics. One month after surgery, a higher relative change in sEV concentration was observed in patients who did not undergo resection compared to those with resected tumors (14.7% vs. 3.1%, respectively), which might indicate a connection of EV concentration to tumor burden, but the difference was not significant. This might be due to the longer time interval after the surgery in our study, as blood samples were collected after one month, while in other studies, which showed a correlation of EV concentration to tumor burden, they were collected up to one week after surgery [28,45,46].

A limitation of our study was a small sample size and relatively short observational period. As only one patient had a well-differentiated tumor, we could not evaluate the association with sEV characteristics for this subgroup. However, we investigated sEV concentration and size in a well characterized population of PDAC patients treated according to the same protocol and in the same institution. Our results should be validated in an independent larger cohort in the future and association of plasma EV characteristics with tumor burden examined in more detail.

In conclusion, plasma sEV concentration and size correlate with tumor differentiation and may predict OS in PDAC patients undergoing surgery with curative intent. Further longitudinal studies on larger study cohorts are needed to evaluate sEVs as composite or complementary biomarkers for preoperative assessment of tumor grade and as prognostic biomarkers for OS, in order to improve patient stratification and treatment optimization. Our study thus complements other innovative approaches in cancer liquid biopsy, such as ctDNA methylation profile and fragmentation [20,21].

## Figures and Tables

**Table 1:** Baseline patients’ characteristics.

Variables ^#^		Study Patients *n* = 34	w/o Resection *n* = 16	With Resection *n* = 18	*p*-Value *
Sex	Male, *n* (%)	21 (61.8)	11 (68.8)	10 (55.6)	0.497 ^c^
	Female, *n* (%)	13 (38.2)	5 (31.3)	8 (44.4)	
Age	Years, median (25–75%)	68.5 (64.8–77.0)	68.5 (65.0–76.5)	67.5 (60.8–77.0)	0.621 ^d^
ASA score	2, *n* (%)	10 (30.3) [1]	5 (31.3)	5 (29.4) [1]	1.000 ^c^
	3, *n* (%)	23 (69.7)	11 (68.8)	12 (70.6)	
Smoking	No, *n* (%)	14 (43.8) [2]	7 (50.0) [2]	7 (38.9)	0.721 ^c^
	Yes, *n* (%)	18 (56.3)	7 (50.0)	11 (61.1)	
Alcohol consumption	None, *n* (%)	9 (28.1) [2]	4 (28.6) [2]	5 (27.8)	0.453 ^c^
	Occasional, *n* (%)	12 (37.5)	7 (50.0)	5 (27.8)	
	Moderate, *n* (%)	10 (31.3)	3 (21.4)	7 (38.9)	
	Heavy, *n* (%)	1 (3.1)	0 (0.0)	1 (5.6)	
BMI ^a^	kg/m^2^, median (25–75%)	24.9 (21.5–28.2)	25.3 (22.4–27.9)	23.3 (21.5–28.6)	0.613
WBC count ^a^	×10^9^/l, median (25–75%)	7.5 (5.6–9.1) [1]	7.5 (5.9–8.7)	7.5 (5.4–9.5) [1]	0.901 ^d^
CRP ^a^	mg/l, median (25–75%)	5 (5–22) [1]	8.5 (5.0–34.3)	5 (5–8.5) [1]	0.102 ^d^
CA 19-9 ^a^	kU/L, median (25–75%)	787.1 (48.0–4568.1)	1967 (61–4699.2)	439.0 (48–4055.5)	0.597 ^d^
CEA ^a^	µg/L, median (25–75%)	4.4 (1.9–8.2)	4.7 (2.1–8.6)	4.4 (1.9–7.5)	0.905 ^d^
Preoperatively evaluatedtumor size	mm, median (25–75%)	30 (25–44.5) [1]	34 (25.8–46.5)	28 (24.5–39) [1]	0.309 ^d^
Borderline resectable	No, *n* (%)	24 (70.6)	9 (37.5)	15 (62.5)	0.134
	Yes, *n* (%)	10 (29.4)	7 (70.0)	3 (30.0)	
Distant metastases ^b^	No, *n* (%)	23 (67.6)	7 (43.8)	16 (88.9)	0.009 ^c^
	Yes, *n* (%)	11 (32.4)	9 (56.3)	2 (11.1)	
Tumor differentiation ^c^	Poor, *n* (%)	14 (45.2) [3]	7 (50.0) [2]	7 (41.2) [1]	1.000 ^c^
	Moderate, *n* (%)	16 (51.6)	7 (50.0)	9 (52.9)	
	Well, *n* (%)	1 (3.2)	0 (0.0)	1 (5.9)	
Adjuvant chemotherapy ^†^	No, *n* (%)	15 (44.1)	8 (50.0)	7 (38.9)	0.730 ^c^
	Yes, *n* (%)	19 (55.9)	8 (50.0)	11 (61.1)	

w/o: without; ASA: American Association of Anesthesiologists; BMI: body mass index; WBC: white blood cell; CRP: C-reactive protein; CA 19-9: carbohydrate antigen 19-9; CEA: carcinoembryonic antigen; [ ]: number of missing data in each category. ^#^ Data collected immediately before surgery (^a^), intraoperatively (^b^) or by definite histology (^c^). ^†^ For 16 (84.2%) patients, adjuvant chemotherapy was initiated more than one month after surgery. Six of those (two without resection, four with resection) additionally received radiation therapy more than one month after surgery. * Comparison between patients w/o resection and patients with resection was calculated using Fisher’s exact test (^c^) or Mann–Whitney test (^d^).

**Table 2:** Patients’ small plasma extracellular vesicle (EV) characteristics.

	Small EV Characteristics	Study Patients *n* = 34 Median (25–75%)	w/o Resection *n* = 16 Median (25–75%)	With Resection *n* = 18 Median (25–75%)	*p*-Value *
Before surgery	Concentration (×10^10^/mL)	6.02 (4.84–7.91)	6.02 (4.83–7.73)	6.10 (5.03–9.03)	0.646
	Mean diameter (nm)	168.1 (157.4–177.2)	165 (155.3–176.2)	173.2 (157.4–178.1)	0.528
	Modal diameter (nm)	136.3 (114.1–150.1)	132.2 (107.8–137.4)	144 (124.3–155)	0.039
	Median diameter (nm)	153.2 (143.8–162.2)	149.8 (144.9–159.9)	157.3 (139.7–165.1)	0.330
After one month	Concentration (×10^10^/mL)	6.46 (6.00–8.40) [7]	7.71 (5.67–15.3)	6.40 (6.05–7.08)	0.359
	Mean diameter (nm)	174.9 (165.3–182.6) [7]	175.9 (152.1–186.9) [6]	174.9 (167.1–182.6) [1]	0.675
	Modal diameter (nm)	133.3 (120.1–153.5) [7]	124.8 (109.9–145.8) [6]	136.5 (125.5–154) [1]	0.286
	Median diameter (nm)	155.7 (150.1–165.9) [7]	156.9 (136–168) [6]	155.7 (154.3–165.9) [1]	0.505
Relative change	Concentration (%)	12.7 (−17.9 do 36.4) [7]	14.7 (−18.3–101.5) [6]	3.1 (−33–31.7) [1]	0.309
	Mean diameter (%)	5.1 (−1.3 do 12.5) [7]	6.7 (−11.9–15.4) [6]	3.9 (0–10.3) [1]	1.000
	Modal diameter (%)	3.6 (−11.1 do 17.9) [7]	7.4 (−17.5–18.1) [6]	−1.5 (−13.1–20.6) [1]	0.902
	Median diameter (%)	4.7 (−2.0 do 12.4) [7]	8.1 (−12.3–13.4) [6]	4.3 (−0.4–11.9) [1]	1.000

w/o: without; [ ]: number of missing data in each category. * Comparison between patients w/o resection and patients with resection was calculated using Mann–Whitney test.

**Table 3:** Association between plasma small EV characteristics and tumor differentiation.

	Small EV Characteristics	Poor Differentiation Median (25–75%)	Well/moderate Differentiation Median (25–75%)	*p*-Value *
Before surgery	Concentration (×10^10^/mL)	5.97 (5.08–7.46)	5.66 (4.53–9.55)	0.984
	Mean diameter (nm)	176.9 (165.9–178.5)	149.2 (144.7–173.6)	0.021
	Modal diameter (nm)	139.8 (130.9–154.2)	135.1 (99.6–143.3)	0.077
	Median diameter (nm)	159.9 (149–165.7)	149.2 (125.0–157.1)	0.023
After one month	Concentration (×10^10^/mL)	6.91 (6.06–10.12)	6.22 (5.10–6.93)	0.096
	Mean diameter (nm)	177.7 (158.5–186.9)	174.2 (169.2–182.5)	0.796
	Modal diameter (nm)	139.9 (113.6–157.9)	129.9 (123.8–143.3)	0.666
	Median diameter (nm)	164.2 (142.7–168.2)	154.7 (153.7–165.2)	0.508
Relative change	Concentration (%)	26.3 (−2.1–71.5)	-3.9 (−35.8 to 20.8)	0.056
	Mean diameter (%)	3.5 (−19.7–6.2)	10.3 (0.2–17.6)	0.056
	Modal diameter (%)	1.1 (−35.1–12.9)	5.7 (−10.7 to 32.9)	0.341
	Median diameter (%)	4.5 (−22.5–9.7)	8.2 (0.1–22.1)	0.192

* Comparison between tumors with poor and well/moderate differentiation was calculated using Mann–Whitney test.

**Table 4:** Association between small EV characteristics and overall survival.

	Small EV Characteristics	HR (95% CI) *	*p*-Value	HR (95% CI)_adj_ *	*p-*Value_adj_
Before surgery	Concentration (×10^10^/mL)	1.00 (1.00–1.00)	0.458	1.00 (1.00–1.00)	0.220
	Mean diameter (nm)	1.03 (0.77–1.36)	0.865	1.10 (0.81–1.50)	0.551
	Modal diameter (nm)	0.95 (0.79–1.14)	0.571	1.08 (0.87–1.34)	0.486
	Median diameter (nm)	0.98 (0.76–1.28)	0.904	1.07 (0.79–1.44)	0.663
Relative change	Concentration (%)	1.11 (0.98–1.27)	0.106	1.25 (1.05–1.50)	0.015
	Mean diameter (%)	0.65 (0.40–1.05)	0.076	0.69 (0.44–1.10)	0.117
	Modal diameter (%)	0.86 (0.68–1.08)	0.197	0.74 (0.57–0.95)	0.019
	Median diameter (%)	0.76 (0.50–1.16)	0.199	0.76 (0.52–1.12)	0.165

HR: hazard ratio; CI: confidence interval; adj: adjusted for CRP levels before surgery and adjuvant chemotherapy. * HR values are reported for a difference of 10 units or 10%.

## Supplementary Materials

The following are available online at https://www.mdpi.com/2075-4426/11/2/77/s1, Table S1: Association between patients’ clinical and plasma small EV characteristics; Table S2: ROC curve analysis to assess the ability of small EV characteristics to discriminate between poorly and well/moderately differentiated tumors.
