# Supplementary material for: Plasma Extracellular Vesicle Characteristics Correlate with Tumor Differentiation and Predict Overall Survival in Patients with Pancreatic Ductal Adenocarcinoma Undergoing Surgery with Curative Intent
Source: J Pers Med. 2021 Jan 28;11(2):77. doi: 10.3390/jpm11020077 (PMC7910876; doi:10.3390/jpm11020077)
Supplement: Supplementary file 1 [file jpm-11-00077-s001.zip › Badovinac D_Supplementary Materials/Supplementary Material.docx]

**Table S1**. Association between patients’ clinical and plasma small EV characteristics

| **Variables**^#^ | **Concentration**  (x 10^10^/ml) | **Mean diameter** (nm) | **Modal diameter** (nm) | **Median diameter** (nm) |
| --- | --- | --- | --- | --- |
| Sex | U=96.5  *p*=0.158 | U=179.5  *p*=0.129 | U=165  *p*=0.326 | U=188.5  *p*=0.065 |
| Age | Spearman's  rho=-0.161 *p*=0.362 | Spearman's rho=0.079 *p*=0.655 | Spearman's  rho=-0.071 *p*=0.688 | Spearman's  rho=-0.053 *p*=0.767 |
| ASA score | U=70  *p*=0.081 | U=167.5  ***p*=0.038** | U=141  *p*=0.324 | U=151.5  *p*=0.155 |
| Smoking | U=144.5  *p*=0.488 | U=122  *p*=0.896 | U=114  *p*=0.667 | U=123.5  *p*=0.925 |
| Alcohol consumption (yes/no) | U=118  *p*=0.564 | U=69  *p*=0.157 | U=67.5  *p*=0.133 | U=70.5  *p*=0.170 |
| BMI^a^ | Spearman's rho=0.344 *p*=0.054 | Spearman's rho=0.025 *p*=0.892 | Spearman's  rho=-0.249 *p*=0.169 | Spearman's  rho=-0.058  *p*=0.754 |
| WBC count^a^ | Spearman's rho=0.330 *p*=0.061 | Spearman's  rho=-0.218 *p*=0.223 | Spearman's  rho=-0.342 *p*=0.051 | Spearman's  rho=-0.236 *p*=0.185 |
| CRP^a^ | Spearman's rho=0.200 *p*=0.266 | Spearman's  rho=-0.134 *p*=0.457 | Spearman's  rho=-0.376 ***p*=0.031** | Spearman's  rho=-0.135 *p*=0.454 |
| CA 19-9^a^ | Spearman's  rho=-0.148 *p*=0.403 | Spearman's rho=0.099 *p*=0.579 | Spearman's  rho=-0.068 *p*=0.704 | Spearman's rho=0.017 *p*=0.926 |
| CEA^a^ | Spearman's  rho=-0.252 *p*=0.150 | Spearman's  rho=-0.281 *p*=0.108 | Spearman's rho=0.084 *p*=0.639 | Spearman's rho=0.229 *p*=0.193 |
| Preoperatively evaluated tumor size | Spearman's  rho=-0.254 *p*=0.154 | Spearman's  rho=-0.185 *p*=0.303 | Spearman's  rho=-0.206 *p*=0.251 | Spearman's  rho=-0.207 *p*=0.248 |
| Distant metastases^b^ | U=107  *p*=0.490 | U=134  *p*=0.800 | U=97.5  *p*=0.291 | U=126  *p*=1.000 |

*ASA* American Association of Anaesthesiologists, *BMI* body mass index, *WBC* white blood cell, *CRP* C-reactive protein, *CA 19-9* carbohydrate antigen 19-9, *CEA* carcinoembryonic antigen

U: Mann-Whitney test statistic

^#^Data collected immediately before surgery (a) or intraoperatively (b)

**Table S2**. ROC curve analysis to assess the ability of small EV characteristics to discriminate between poorly and well/moderately differentiated tumors

|  | **Small EV characteristics** | **AUC** (95% CI) | ***p-*Value** | **Cutoff*** | **Sensitivity** | **Specificity** |
| --- | --- | --- | --- | --- | --- | --- |
| Before surgery | Concentration  (x 10^10^/ml) | 0.504 (0.294-0.714) | 0.968 | 4.83 | 0.353 | 0.857 |
|  | Mean diameter (nm) | 0.742 (0.560-0.923) | **0.022** | 173.55 | 0.765 | 0.714 |
|  | Modal diameter (nm) | 0.689 (0.502-0.876) | 0.074 | 151.00 | 0.941 | 0.357 |
|  | Median diameter (nm) | 0.737 (0.558-0.917) | **0.025** | 158.85 | 0.824 | 0.643 |
| Relative change | Concentration (%) | 0.736 (0.529-0.942) | 0.053 | 33.82 | 0.929 | 0.500 |
|  | Mean diameter (%) | 0.736 (0.534-0.938) | 0.053 | 11.37 | 0.500 | 1.000 |
|  | Modal diameter (%) | 0.621 (0.389-0.854) | 0.320 | -28.03 | 1.000 | 0.300 |
|  | Median diameter (%) | 0.664 (0.442-0.886) | 0.178 | 15.28 | 0.357 | 1.000 |

*AUC* area under the curve, *CI* confidence interval

*Cutoff with the highest sum of specificity and sensitivity
